# Radiation-Induced Normal Tissue Damage: Oxidative Stress and Epigenetic Mechanisms

**DOI:** 10.1155/2019/3010342

**Published:** 2019-11-12

**Authors:** Jinlong Wei, Bin Wang, Huanhuan Wang, Lingbin Meng, Qin Zhao, Xinyu Li, Ying Xin, Xin Jiang

**Affiliations:** ^1^Department of Radiation Oncology, The First Hospital, Jilin University, Changchun 130021, China; ^2^Department of Internal Medicine, Florida Hospital, Orlando, FL 32803, USA; ^3^Key Laboratory of Pathobiology, Ministry of Education, Jilin University, Changchun 130021, China

## Abstract

Radiotherapy (RT) is currently one of the leading treatments for various cancers; however, it may cause damage to healthy tissue, with both short-term and long-term side effects. Severe radiation-induced normal tissue damage (RINTD) frequently has a significant influence on the progress of RT and the survival and prognosis of patients. The redox system has been shown to play an important role in the early and late effects of RINTD. Reactive oxygen species (ROS) and reactive nitrogen species (RNS) are the main sources of RINTD. The free radicals produced by irradiation can upregulate several enzymes including nicotinamide adenine dinucleotide phosphate oxidase (NADPH oxidase), lipoxygenases (LOXs), nitric oxide synthase (NOS), and cyclooxygenases (COXs). These enzymes are expressed in distinct ways in various cells, tissues, and organs and participate in the RINTD process through different regulatory mechanisms. In recent years, several studies have demonstrated that epigenetic modulators play an important role in the RINTD process. Epigenetic modifications primarily contain noncoding RNA regulation, histone modifications, and DNA methylation. In this article, we will review the role of oxidative stress and epigenetic mechanisms in radiation damage, and explore possible prophylactic and therapeutic strategies for RINTD.

## 1. Introduction

Cancer is one of the most challenging diseases in modern times. In 2015, China reported about 4.2 million new cancer cases and 2.8 million cancer-related deaths [1]. Radiotherapy (RT) is currently one of the leading therapeutic approaches for several cancers; however, it carries the potential to cause injury to normal tissue, with both short-term and long-term side effects. In recent years, studies have shown that the oxidation/reduction (redox) system was associated with several types of damage after radiation exposure [2]. In addition, the redox system is related to epigenetic regulation and can regulate the expression of microRNAs (miRNAs) and other molecules, thus playing a role in sustained oxidative damage after radiation [3].

Cells and tissues are composed of about 80% or more water, and most of the radiation damage occurs due to the radiolysis of water, which induces the production of reactive oxygen species (ROS) and reactive nitrogen species (RNS) [4]. ROS and RNS are the main sources of radiation-induced normal tissue damage (RINTD). The generation of ROS induces molecular changes and causes oxidative damage to proteins, lipids, and DNA. It can also activate signal transduction pathways and early-response transcription factors [5]. The redox system plays an important role in acute radiation damage and is responsible for some radiation-induced early and late effects including inflammation, out-of-field effects, fibrosis, bystander effects, and others [6–9].

In recent years, several studies have demonstrated that epigenetic modulators play an important role in normal tissue damage, after redox-induced ionizing radiation. Epigenetic modifications are made up of the heritable changes in the expression of the gene that do not influence the sequence of the DNA. In mammals, epigenetic modifications primarily contain noncoding RNA regulation, histone modifications (methylation, phosphorylation, and acetylation), and DNA methylation. Epigenetic changes can be reversible and can easily respond to natural bioactive dietary compounds [10]. Afanas'ev et al. has reported that free radicals such as NO and ROS can regulate and control the epigenetic processes [11]. In addition, the regulation of some miRNAs may decrease or increase the oxidative damage [11].

In regard to the damage caused by RT, treatment strategies are still lacking. Here, we review the role of oxidative stress and epigenetic mechanisms in radiation damage to explore possible therapeutic strategies for RINTD.

## 2. Oxidative Stress

**Figure 1:**
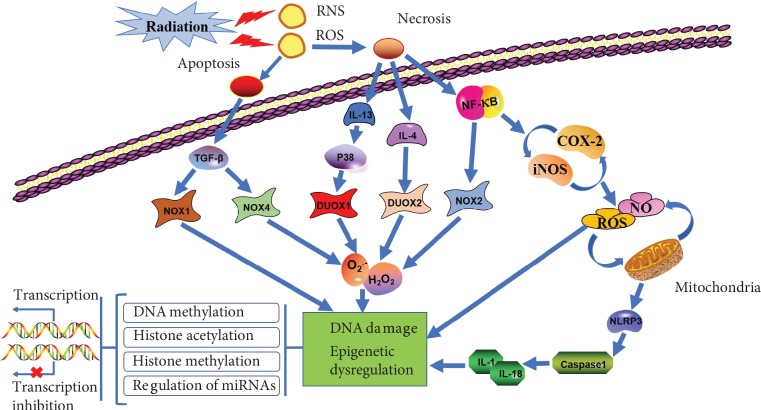
The mechanisms of redox system activation, inflammation response, and epigenetic regulation following exposure to radiation.

Oxidative stress is involved in the development of many diseases including RINTD. The redox system plays an important role in the early and late effects of RINTD [12]. When cells are exposed to radiation, they immediately form free radicals with a half-life of nanoseconds. The redox system begins producing free radicals a few hours after exposure, with the potential to last for years [13, 14]. The free radicals produced by ionizing radiation can upregulate several enzymes, including nicotinamide adenine dinucleotide phosphate oxidase (NADPH oxidase), lipoxygenases (LOXs), nitric oxide synthase (NOS), and cyclooxygenases (COXs). Their effects on mitochondrial function are distinct. These enzymes are expressed in specific ways in various cells, tissues, and organs (Figure 1).

### 2.1. NADPH Oxidases

NADPH oxidase (NOX) is thought to be a membrane-bound oxidoreductase. It can transfer electrons from NADPH to the oxygen molecules. In addition, some subtypes of these enzymes have been found in cells [12]. NADPH oxidase enzymes such as DUOX1, DUOX2, and NOX1-5 are the most crucial subtypes. They participate in the process of respiratory chain rupture after radiation [15]. These enzymes have the ability to transfer electrons across the plasma membrane and produce superoxide and other downstream ROS. However, the tissue distribution and activation mechanisms of the individual members of the NOX family are undoubtedly different [16]. In addition, many inflammatory cytokines and chemokines such as TGF-*β*, TNF-*α*, IL-1, and IFN-*γ* are involved in the NOX system activation [17]. NADPH oxidase enzymes play a key role in acute and chronic oxidative stress in bystander and directly irradiated cells [18]. Also, it has been shown that the expression of NOX2 and NOX4 can be upregulated in nontargeted tissues [19].

NOX1 is the first homolog of NOX2 described (then called gp91^phox^) [20, 21]. NOX1 can be expressed in a variety of cells including endothelial cells in the uterus, prostate, and placenta, as well as osteoclasts. It can also be expressed in some malignant tumors including colon cancer and melanoma [22–24]. Choi et al. reported that the NOX1-specific inhibitor can limit radiation-induced collagen deposition and fibroblastic changes in the endothelial cells, thereby alleviating pulmonary fibrosis induced by radiation [25]. In addition, the production of ROS was significantly decreased after inhibition of NOX1.

NOX2 is considered the prototype of the NADPH oxidase. A report by Kim et al. confirmed that NOX2 was involved in radiation-induced salivary gland damage. After 56 days of exposure to 18 Gy radiation, the expression of the NOX2 gene in the salivary glands of rats was significantly increased. In addition, the apoptotic genes such as caspase-9 and MAPKs, including p-38 and JNK, participate in NOX2 signaling cascades [26]. Experiments conducted by Narayanan et al. demonstrated that irradiation of human lung fibroblasts generated O_2_^•-^ and H_2_O_2_ with alpha particles. The plasma membrane-bound NOX2 is responsible for the production of O_2_^•-^ and H_2_O_2_ [27]. Datta et al. confirmed that long-range mitochondrial dysfunction and the increased NADPH oxidase, including NOX1 and NOX2 activity, are the main factors for radiation-induced continuous oxidative stress in the intestinal epithelial cells [28].

NOX3 was first described in the year 2000 based on its similarity to the sequences of other NOX subtypes [29], although the function of the protein was first studied in 2004 [30, 31]. At present, the study of NOX3 in radiation damage is limited. Shin et al. confirmed that the expression of NOX3 was upregulated after the irradiation of the oral mucosa of rats. The increased expression of NOX3 is thought to be related to the necrotic inflammatory exudate and oral mucosa ulcers [32].

NOX4 was initially thought to be an NADPH oxidase homolog, highly expressed in the kidney [33, 34]. Pazhanisamy et al. found that systemic irradiation in mice can selectively induce sustained oxidative stress in hematopoietic stem cells (HSCs), at least in part, by the upregulation of NOX4. The increased production of ROS by NOX in HSCs can mediate radiation-induced hematopoietic genomic instability [35]. The experimental results of Wang et al. showed that systemic irradiation selectively induces chronic oxidative stress in HSCs, at least in part by the upregulation of NOX4 expression, thereby giving rise to the induction of HSC senescence and residual bone marrow damage [36]. In addition, the TGF-*β*-NOX4 pathway may be responsible for the continuous production of ROS/NO and the subsequent genomic instability after bone marrow irradiation [37].

NOX5, found in the lymphoid and testis, contains an N-terminal extension with three EF hands, and it can produce superoxide dismutase and conduct H^+^ ions when intracellular free Ca^2+^ increases [38–40]. NOX5 may participate in Ca^2+^-activated, redox-dependent processes of spermatozoa and lymphocytes including cytokine secretion, cell proliferation, and sperm-oocyte fusion [41]. Weyemi et al. showed that the two members of NADPH oxidase, NOX4 and NOX5, participated in the process of radiation-induced DNA damage in human primary fibroblasts.

Currently, there is a small amount of evidence supporting the role of DUOX1/DUOX2 in chronic oxidative stress. Both DUOX1 and DUOX2 are highly expressed in the thyroid gland [42, 43]. Furthermore, DUOX1 can be found in the prostate and airway epithelia [44–47]. DUOX2 has been described in the salivary gland, airway epithelia, prostate, rectal mucosa, duodenum, cecum, and colon [44–49]. Ameziane-El-Hassani et al. demonstrated that radiation-induced DUOX1-dependent H_2_O_2_ production by NADPH oxidase was delayed in a dose-dependent manner for several days. In addition, p38 MAPK, which was activated after irradiation, can increase DUOX1 through the expression of IL-13, giving rise to sustained DNA damage and growth stagnation [50]. Wu and Doroshow showed that IL-4/IL-13 can induce the expression of DUOX2/DUOXA2 and the production of ROS in human colon and pancreatic cancer cells, which in turn may be related to the occurrence of inflammatory gastrointestinal malignancies [51]. There are some studies that have shown the upregulation of DUOX1 and DUOX2 in the lung and heart. Some radioprotectors and antioxidants such as melatonin, metformin, and selenium have been shown to reduce the expression of these genes following exposure to ionizing radiation, relieving the heart damage and lung damage caused by radiation [52–56]. However, the relationship between DUOX2 expression and radiation-induced carcinogenesis has not been established and demands further verification.

### 2.2. COX-2

Cyclooxygenase-2 (COX-2), an isoform of cyclooxygenase, is responsible for the time-dependent and localized production of prostaglandins (PGs) at inflammatory sites [57], including tissues exposed to ionizing radiation. COX-2 plays a crucial role in the inflammatory response that converts arachidonic acid released by membrane phospholipids into PGs. In addition, ROS production is a standard secondary byproduct of arachidonic acid metabolism in the synthesis of PGE2 [58]. Several studies have shown that increased COX-2 expression is related to radiation toxicity after the irradiation of organs, such as the lungs, heart, kidneys, intestines, colon, and the brain [59, 60]. Other studies have reported that COX-2 is involved in the pathogenesis of vascular damage, atherosclerosis, and fibrosis induced by ionizing radiation [61]. Cheki et al. demonstrated that celecoxib, an inhibitor of COX-2, can decrease dermal inflammation, MCP-1 mRNA expression, and radiation-induced skin reactions [62]. In addition, several studies have investigated the role of nonsteroidal anti-inflammatory drugs (NSAIDS) as an inhibitor of COX on radiation damage in the lung and joints [63–65]. Clinically approved inhibitors are represented by NSAIDs like aspirin or ibuprofen and by selective COX-2 inhibitors such as celecoxib [60].

### 2.3. LOXs

LOXs are enzymes that dioxygenate unsaturated fatty acids, which can initiate lipoperoxidation of the membrane, synthesize signaling molecules, or induce cell structural and metabolic changes [66]. Currently, the role of LOX in radiation damage has been reported. Matyshevskaia et al. demonstrated that activated LOX is involved in the production of ROS after exposure and plays an important role in the process of radiation-induced DNA fragmentation in lymphocytes [67]. Another experimental study showed that LOX was activated immediately after exposure to thymocytes. High LOX activity was observed in cells within an hour after irradiation. In addition, radiation-induced generation of lipid peroxides may be a factor in LOX activation [68]. In another study, Halle et al. showed that chronic adventitial inflammation, vasa vasorum expansion, and 5-LOX upregulation are involved in radiation-induced arterial damage in cancer survivors. In previously irradiated arterial segments, the expression of 5-LOX was increased in exogenous macrophages surrounding vascular dilatation [69].

### 2.4. Nitric Oxide

Under conditions of stress, including inflammation, inducible nitric oxide synthase (iNOS) was thought to be the primary source of nitric oxide (NO) and played an important role in carcinogenesis and the oxidative stress process. NO is generated by macrophages under the stimulation of inflammation through the iNOS enzyme, and it can interact with the mitochondria-derived superoxide to further produce peroxynitrite [70]. iNOS enzymes play a key role in the radiation damage via posttranslational regulation of the BER pathway of DNA repair. The main effect of NO is nitroacetylation of 8-xoguanine glycosylase (Ogg1). Ogg1 inhibition by NO can result in increased accumulation of oxidative DNA lesions [71, 72]. Malaviya et al. noted that iNOS is involved in radiation-induced lung damage. In addition, there are complex interactions between oxidative and nitrosative stress, as well as inflammatory pathways that mediate lung damage after radiation [73]. In another study, the inhibitors of iNOS such as aminoguanidine and N-nitro-L-arginine methyl ester have been shown to reduce radiation-induced lung damage [74, 75]. In a study by Ohta el al., increased levels of NO were directly related to the radiation dose, and NO levels increased in the first few hours after receiving the radiation [76].

The role of NO in the radiation-induced bystander effect has been explored. The peculiarity of NO as a redox signaling molecule is partly due to its hydrophobic properties and relative stability [77]. The hydrophobicity of NO allows it to diffuse through the cytoplasm and plasma membrane, allowing this kind of signaling molecule to readily diffuse from irradiated cells to bystander cells without the involvement of gap junction intercellular communication. NO generated in the irradiated tissues can mediate cellular regulation through posttranslational modification of many regulatory proteins [78]. Ghosh et al. have shown that activated iNOS in irradiated cells are crucial to the bystander response. In addition, lipopolysaccharide-induced iNOS activity and the production of NO after irradiation increased bystander cell DNA damage [79, 80]. Han et al. showed that within 30 min of low-dose alpha-particle irradiation, NO played an important role in the process of DNA double-strand breaks in bystander cells [81].

### 2.5. Oxidative Stress and Inflammation

Inflammatory responses are thought to play an important role in redox activation. Normal cells that are directly exposed to irradiation or ROS will give rise to nuclear and mitochondrial DNA damages, which can lead to cell death via processes such as mitotic catastrophe, apoptosis, and necrosis [82]. Necrosis can trigger the release of inflammatory cytokines such as IL-1, IL-4, IL-13, and other inflammatory mediators, while apoptosis may cause the release of anti-inflammatory cytokines including TGF-*β* and IL-10 [83, 84]. ROS are the main cause of RINTD. The continuous formation of ROS after IR exposure can be the source of radiosensitivity of the T lymphocytes and other cells [85]. Moreover, ROS can activate the NF-*κ*B signaling pathway along with proinflammatory cytokines. NF-*κ*B plays a key role in chronic inflammatory diseases after RT [86]. Inflammatory cytokines and growth factors can give rise to a variety of signaling cascades, such as NADPH oxidase, COX-2, and iNOS [87]. It has also been reported that COX-2 is an important gene which can mediate the subsequent inflammatory responses [88]. Mitochondria is thought to be an energy and free radical reservoir. In normal conditions, antioxidant defense systems neutralize superoxide and form free radicals and protect cells from oxidative damage resulting from mitochondrial activity [89]. However, mitochondrial dysfunction and apoptosis can be induced by ROS, pro-IL-1*β*, iNOS, and inflammatory responses. Also, studies have shown that the ROS-derived NOX system participated in mitochondrial dysfunction and in the subsequent production of ROS in this organelle [90]. Next, the damaged mitochondria will release ROS and activate the nucleotide-binding domain and the leucine-rich-repeat-containing family pyrin 3 (NLRP3) inflammasome pathway [91]. The activation of the NLRP3 inflammasome is the platform of caspase-1 activation. Finally, it will lead to the secretion of proinflammatory cytokines IL-18 and IL-1*β* [92]. Recent experiments and studies have shown that the upregulation of the NLRP3 inflammasome has a big impact on radiation damage [93–96]. Chronic inflammatory processes can exist for ages after irradiation, and the immune system does not suppress them. This is related to chronic oxidative damage giving rise to the genomic instability and impaired normal tissue function [97].

### 2.6. Oxidative Stress and Cellular Senescence

Cellular senescence can be induced by ionizing radiation. Radiation-induced senescence is mainly one of the mechanisms in radiation-induced pathological changes. Radiation-induced cellular senescence is thought to be caused by p53 activation, which is associated with a radiation-induced double-strand DNA break [98]. However, the exact mechanism of inducing cellular senescence is still unclear, but the involvement of ROS has been widely reported [99, 100]. The study of Kobashigawa et al. suggested that the delayed increase of intracellular oxidative stress levels plays a key role in the process of radiation-induced cellular senescence by p53 accumulation [101]. Sakai et al. showed that NOX4 can mediate the production of ROS in radiation-induced senescent cells and lead to normal tissue damage after irradiation through recruitment of inflammatory cells and intensification of tissue inflammation [102].

## 3. Epigenetic Mechanisms

### 3.1. Epigenetics and Cancer

Cancer is commonly thought to be caused by genetic alterations including deletions, insertions, mutations, recombination, copy number gains, single-nucleotide polymorphisms, and genomic instability [103, 104]. The latest evidence suggests that cancer may occur without changes in the nucleotide sequence, by means of alleged epigenetic alterations. Combinational crosstalk between epigenetic alterations and genetics has been known to play a role in the development, progression, and recurrence of cancer [105]. Miousse et al. reported that epigenetic alterations are among the driving forces of irradiation-induced carcinogenesis, by observing long interspersed nucleotide element 1 DNA methylation changes in the mouse hematopoietic system after irradiation [106].

Epigenetic dysregulation including increased activity of histone deacetyltransferases (HDACs), DNA methyltransferases (DNMTs), and changes in the noncoding RNA expression, can give rise to changes in gene transcription and expression, which regulate cell cycle, cell differentiation and proliferation, and apoptosis [107, 108]. Yi et al. showed that the combined action of DNMT and HDAC inhibitors could stagnate the cell cycle at the G2/M phase and suppress the growth of endometrial cancer by upregulating E-cadherin and downregulating Bcl-2 [107]. Choi et al. noted that DNMTs including DNMT3A, DNMT3B, and DNMT1 are overexpressed in the hepatocellular carcinoma compared with noncancerous liver samples [109]. One such study has demonstrated that HDAC5 can promote glioma cell proliferation by upregulating the expression of Notch 1 at both the mRNA and the protein level [110]. In addition, HDAC5 can also promote human HCC cell proliferation by upregulating the expression of Six1 [111]. Epigenetic regulation, as a molecular target for cancer prevention and therapy, has aroused wide interest. For example, some studies showed that (-)-epigallocatechin-3-gallate, a main component of green tea, could possibly bind with the DNMTs, reducing the methylation activity of cancer cells through epigenetic mechanisms, and thus leading to cancer prevention or treatment [112, 113]. At present, there is increasing evidence that targeting epigenetic modifications is an effective cancer prevention strategy.

### 3.2. Epigenetics and RINTD

In recent years, the relationship between epigenetic mechanisms and radiation damage has been studied extensively [114–116]. Currently, epigenetic mechanisms such as DNA methylation and miRNA and histone modifications are reported to be associated with radiation damage. These mechanisms are summarized in Table 1.

#### 3.2.1. DNA Methylation

DNA methylation is a crucial means of epigenetic modification, which primarily occurs in the CPG islands of the gene promoter regions. Multiple DNMT functions are required to establish and maintain DNA methylation patterns [117]. Therapeutic radiation can give rise to biological responses to confront the subsequent DNA damage and genomic stress, to avoid cell death. Antwih et al. showed that the DNA methylation response to radiation is parallel to the classical biological responses to radiation. The differential methylation level of DNA repair, cell cycle, and apoptosis pathways varied with different radiation doses [118]. Fractionated low-dose radiation exposure has been reported to cause the accumulation of DNA damage and profound alterations in DNA methylation in the murine thymus. This could be the source of the risk of radiation-induced leukemia and thymic lymphoma [119].

Acharya et al. showed that neuroepigenetic mechanisms played an important role in affecting the functional and structural changes in the brain and in cognition after irradiation. In irradiated mice with cognitive impairment, 5-hydroxymethylcytosine and 5-methylcytosine were detected in the region of the hippocampus consistent with increased levels of Ten Eleven Translocation- (TET-) 1, TET3, and DNMT3a. DNMT3a and TET enzymes including TET1 and TET3 are related to addiction behavior and memory formation. In addition, they found an obvious improvement in the epigenetic effects of irradiation by inhibiting methylation using 5-iodotubercidin [116]. Koturbash et al. demonstrated the role of epigenetic effects in maintaining the long-term, persistent bystander effect in the spleen, in vivo. After localized cranial irradiation for 24 h and 7 months, the levels of methyltransferases DNMT3a, DNMT3b, and DNMT1 and methyl-binding protein MeCP2 in the spleen tissue were significantly decreased [120].

The above results indicate that radiation can cause changes in DNA methylation to modify and regulate the expression of related genes and proteins, thus causing the corresponding tissue and organ damage. Future research is essential to confirm the role of DNA methylation in radiation-induced normal tissue damage. In addition, DNA methylation can be used as a target to prevent and treat radiation damage in the future.

#### 3.2.2. Histone Modifications

Histone modification is rarely studied in radiation-induced normal tissue damage. Most reports have focused on the regulatory role of histone modification in radiation approaches for killing tumor cells [121–123]. Histone modifications include methylation, phosphorylation, and acetylation. Herberg et al. showed that mismatch repair-deficiency leads to genome-wide changes in histone H3 methylation profiles prior to tumorigenesis. Analogous changes constitute a lasting epigenetic feature of radiation-induced DNA damage [124]. Zhang et al. showed that solar-simulated ultraviolet radiation can influence both histone acetyltransferase and HDAC activities causing decreased histone acetylation. This could be the main cause for radiation-induced skin DNA damage [125]. Further research is needed to verify the role of histone modifications in radiation damage.

#### 3.2.3. Regulation of miRNAs

MiRNAs combined with mRNAs can lead to posttranscriptional degradation or repression [126]. It is well known that the role of miRNAs in ROS production and oxidative stress is to increase the superoxide level by suppressing antioxidant enzymes. A good example is the upregulation of miR-21 in both targeted and bystander cells. MiR-21, which is triggered by TGF-*β*, can inhibit SOD2 gene expression, giving rise to a decrease in the activity of SOD2 and damage to irradiated and bystander cells by superoxide [3, 127]. In addition, the SOD activity and glutathione level were inhibited which have been revealed in nontargeted lung tissues [128]. Many studies have shown a link between miRNA regulation and RINTD. miRNAs can play an important role in the early evaluation of radiation-induced hematopoietic damage, as functional dosimeters of radiation [129, 130]. Li et al. also demonstrated that miR-30c plays a key role in radiation-induced hematopoietic and osteoblast cell damage, possibly by regulating the expression of the gene REDD1 [131].

Another study by Li et al. reported that the isomer of vitamin E, delta-tocotrienol, can inhibit radiation-induced miR-30 and protect human and mice CD34^+^ cells from radiation damage by inhibiting IL-1*β*-induced NF-*κ*B/miR-30 signaling [132].

Radiation-induced lung damage includes chronic fibrosis and acute pneumonia [133]. miRNAs have been reported in many diseases including those with lung involvement [134]. Gao et al. showed that miR-19a-3p, miR-144-5p, and miR-144-3p are upregulated in rats 2 weeks after thorax irradiation [135]. Recently, Xie et al. also studied the response of lung miRNA expression to radiation-induced lung damage in rats [136]. MiRNAs may serve as biomarkers for early stages of radiation-induced lung damage.

Radiation-induced spleen damage has also been reported in recent years. Ghosh et al. reported that whole-body radiation exposure resulted in higher expression of miRNAs in the spleen tissue on day 4 and on day 250. In addition, the vitamin E analog gamma-tocotrienol can modulate radiation-induced miRNA expression in the mouse spleen, preventing radiation damage to the spleen [137].

Collectively, miRNAs can serve as promising candidates for radiation biodosimetry. In addition, the prevention and treatment of RINTD through miRNA regulation may have a promising future.

## 4. Conclusions

In summary, oxidative stress responses and epigenetic mechanisms play important roles in RINTD. The redox system and various oxidases upregulated by free radicals and generated by radiation, including NADPH oxidase, LOXs, NOS, and COXs, participated in RINTD through different regulatory mechanisms. ROS and NOS produced by inflammatory cells and mitochondria are involved in oxidative damage to bystander cells and untargeted tissues. In addition, a variety of inflammatory factors including the NLRP3 inflammasome play an important role in radiation-induced oxidative stress damage. Epigenetic mechanisms such as DNA methylation, regulation of miRNAs, and histone modifications have been extensively studied in recent years in relation to RINTD. New progress has been made in the field of radiation damage treatment through the regulation of epigenetic mechanisms. With a further understanding of oxidative stress and epigenetic regulatory mechanisms, we hope to better explore the preventive and therapeutic strategies in RINTD in the future.

## Figures and Tables

**Table 1:** The epigenetic regulation in radiation-induced normal tissue damage.

Epigenetic mechanisms	Irradiation organ	Epigenetic functions	Target genes/proteins	Damage effects	Reference
DNA methylation	Brain	Increased expression of DNMT1 and 3a	Increased expression of TET1 and TET3 proteins	Radiation-induced cognitive dysfunction	Acharya et al. [116]
	Thymus	Decreased expression of DNMT1, 3a, and 3b	Decrease in the levels of methyl-binding proteins MeCP2 and MBD2	Increased the risk of radiation-inducedleukemia and thymic lymphoma	Pogribny et al. [119]
	Human breast cancer cells (MDA-MB-231)	Decreased DNMT1 expression	Downregulation of RB1 expression	DNA damage and apoptosis	Antwih et al. [118]
	Brain	Decreased expression of DNMT1, 3a, and 3b	Decrease in the levels of methyl-binding protein MeCP2	Bystander effect in the spleen	Koturbash et al. [120]

Histone methylation	Intestine	Increased expression of histone H3 methylation	—	Radiation-induced intestinal damage	Herberg et al. [124]

Histone acetylation	Skin	Inhibition of HDAC activity	—	Radiation-induced skin damage and carcinogenesis	Zhang et al. [125]

Regulation of miRNAs	Hematopoietic system	Upregulation of miR-30a-3p, miR-30c-5p, etc.	—	Radiation-induced hematopoietic damage	Acharya et al. [129]
	Lung	Upregulation of miR-19a-3p, miR-144-5p, and miR-144-3p	—	Radiation-induced lung injury	Gao et al. [135]
	Spleen	Increased expression of miR-34a	Upregulation of gene p53	Radiation-induced spleen damage	Ghosh et al. [137]
	Hematopoietic and osteoblast cells	Increased expression of miR-30c	Suppression of gene REDD1	Radiation-induced hematopoietic and osteoblast cell damage	Li et al. [131]
